# A survey of patient compliance with removable orthodontic retainer wear in Brunei Darussalam

**DOI:** 10.1038/s41405-023-00138-8

**Published:** 2023-03-03

**Authors:** Meng Ern Lim, Jagjit Singh Dhaliwal, Siti Waznah Hj Abd Wahab, Hanif Abdul Rahman

**Affiliations:** 1 PAPRSB Institute of Health Sciences, Universiti Brunei Darussalam, Jalan Tungku Link, Gadong, BE1410 Brunei Darussalam; 2 Orthodontics Unit, Department of Dental Services, Ministry of Health, Jalan Lorong 1 Barat, Seria, KB1133 Brunei Darussalam

**Keywords:** Orthodontics, Dental treatments

## Abstract

**Objective:**

To explore compliance with removable orthodontic retainer wear among patients who had completed fixed appliance orthodontic treatments.

**Materials and methods:**

A cross-sectional online survey was distributed to patients who had completed orthodontic treatment at the government orthodontic clinics. The response rate was 54.9%, 663 questionnaires were distributed and 364 responses were received. Demographic information was collected, and questions regarding types of retainers prescribed, instructed and actual wear times, satisfaction levels, and reasons for wearing and not wearing retainers. Chi Square, Fisher’s Exact tests and Independent T-Test were used to detect significant associations between variables.

**Results:**

Respondents under 20 years old and employed were the most compliant. The mean satisfaction levels of Hawley Retainer and Vacuum-Formed Retainer were reported to be 3.7 (*P* = 0.565). About 28% of those in both groups stated that they wear them to maintain their teeth straight. 32.7% of Hawley retainer wearers reported not wearing their retainers due to speech difficulties.

**Conclusion:**

Age and employment status were the variables that determined compliance. There was no significant difference in the satisfaction levels between the two retainer types. Most respondents wear their retainers to keep their teeth straight. Discomfort and forgetfulness were the primary reasons for not wearing retainers, besides speech difficulties.

## Introduction

Orthodontic treatments are only considered successful when the treatment goal is achieved and the results remain stable [1]. Successful orthodontic outcomes are mainly based on retention to prevent relapse [2]. Therefore, retention is of paramount importance as it provides stability to the newly positioned teeth, resulting in improved aesthetics and functional occlusion [3].

Retention of the post-treatment position of the teeth can be achieved by orthodontic retainers, which can be fixed or removable. Fixed retainers can be bonded to the lingual surface of the canines only or bonded to each tooth from canine to canine [4]. On the other hand, several types of removable retainers are available, such as Hawley’s retainer, Begg’s Retainer and Clip-on/Spring Retainer [5]. However, when removable retainers are indicated, patient compliance is an issue [6].

A patient’s overall compliance has been reported to be affected by several factors, such as the socioeconomic and demographic factors, educational level, doctor-patient relationship, general information about treatment, family background, regimen and comfort, the influence of the treatment provider and parental guidance [7]. According to Vig [8], several significant factors identified to be associated with patient’s compliance in the retention phase were age, gender, type of retainer and time since the removal of the fixed appliance. However, Kacer [9] reported that the patients’ age, gender and types of retainers did not affect the levels of compliance. Besides that, other reasons which have been identified were eating (84.3%), speech (56.9%), comfort (47.1%), and both retainers affected participant’s breath odour (43.1%) [10].

Due to the lack of evidence on retention, it leaves orthodontists with several different viewpoints and practice guidelines. Retention protocol is mainly based on the information and knowledge acquired by the orthodontists during residency or clinical experience [11]. Moreover, according to Andriekute et al. [12], orthodontists have no consensus on the necessity for retention, the types of retainers to use, or the length of time retainers should be worn after completing orthodontic treatment.

Since this type of study on removable orthodontic retainers has yet to be conducted in Brunei Darussalam, it will benefit orthodontists in having the proper guidelines in the prescription of removable orthodontic retainers. This study evaluated the wear times between the three removable orthodontic retainers’ wearers (Hawley retainers only, Vacuum-formed retainers only and those wearing a combination of Hawley and Vacuum-Formed retainers), explored the association of the difference in the actual wear time with demographic factors, assessed the satisfaction levels between the two removable orthodontic retainers, and the reasons for low patients’ satisfaction.

## Materials and methods

### Study design and population selection

This is a cross-sectional study that was conducted from July 2021 to May 2022. The participants were patients who have completed fixed appliance orthodontic treatments from all government orthodontic clinics. The inclusion criteria comprised patients above 18 years old who have started and completed fixed appliance orthodontic treatments with National Dental Centre, Berakas Health Centre, Pengiran Muda Mahkota Pengiran Muda Haji Al-Muhtadee Billah Tutong Hospital and the Seria Health Centre. The exclusion criteria are patients who have had upper or lower active removable orthodontic appliances treatment only, retainers other than Hawley retainers and Vacuum-formed retainers, patients with bonded fixed retainers and emergency cases from private clinics as patients do not return for follow-up.

### Data collection and research instrument

Upon approval by the Institute of Health Sciences Research Ethics Committee, Universiti Brunei Darussalam, and Ministry of Health Research and Ethics Committee, online questionnaires (Appendix 1) were distributed to patients who have fulfilled the inclusion criteria. The questionnaire was adapted from the survey used in a study conducted by Sawhney [13] as it covered most of the objectives of this proposed study. The questionnaire was pre-tested among 7 participants from Seria Health Centre. These participants were not included in the main study.

### Sampling and sample size

A sample size of at least 324 is required to achieve precision (power) of 5% (*d* = 0.05) on a population size of 1176 orthodontic retainer patients with an expected proportion of 50% at a 95% confidence level. Accounting for attrition and missing data, a total of 400 questionnaires were targeted to be filled (Naing et al. [14]).

### Data analysis

Information was entered and analysed using RStudio Desktop Version 1.3.1093 (for Mac). The statistical analyses included descriptive and correlational inferential analysis, including one-way ANOVA and Chi-square test for independence, to determine the relationship between the actual retainer wear time and study factors, which includes gender, age, employment status, highest level of education, year braces removed and type of retainers. All statistical tests were two-sided, and a p-value less than 0.05 was considered significant.

## Results

A total of 663 questionnaires were distributed, and 364 responses were received (54.9%). After removing responses with incomplete answers and responses of individuals declining to participate, only 307 responses were accepted (response rate = 46.3%). Table 1 shows the demographic characteristics of the participants, including the retainer types used by them, and the patient compliance to retainer wear according to these characteristics. Most of the respondents were female (80.8%). Over two-third of the participants were aged between 20 and 30 years (69.1%), whereas only 1.6% of respondents were over 40 years. Slightly over half of the respondents were employed, and almost one-third were students. Most of the respondents have tertiary education as their highest level of education. Around 26% of the participants removed their braces in 2019 and 2020, respectively. Vacuum-Formed retainer was the most-prescribed removable retainer (48.9%).

**Table 1:** Patient compliance to retainer wear according to demographic factors (*n* = 307).

	Compliance		Total	*P* Value
	Compliant (%)	Non-compliant (%)		
Gender				
Male	41 (69.5)	18 (30.5)	59 (19.2)	0.143
Female	144 (58.1)	104 (41.9)	248 (80.8)	
Age Group (years old)				
<20	31 (63.3)	18 (36.7)	49 (16.0)	<0.001ª^, *^
20–30	127 (59.9)	85 (40.1)	212 (69.1)	
30–40	24 (58.5)	17 (41.5)	41 (13.4)	
>40	3 (60.0)	2 (40.0)	5 (1.6)	
Employment Status				
Employed	99 (62.7)	59 (37.3)	158 (51.5)	<0.001ª^, *^
Unemployed	25 (59.5)	17 (40.5)	42 (13.7)	
Self-Employed	3 (42.9)	4 (57.1)	7 (2.3)	
Studying	58 (58.0)	42 (42.0)	100 (32.6)	
Highest Level of Education				
Secondary School	21 (65.6)	11 (34.4)	32 (10.4)	0.635
Pre-University	15 (53.6)	13 (46.4)	28 (9.1)	
Tertiary Education	149 (60.3)	98 (39.7)	247 (80.5)	
Year Braces Removed				
2018	44 (62.9)	26 (37.1)	70 (22.8)	0.499
2019	50 (62.5)	30 (37.5)	80 (26.1)	
2020	43(53.1)	38 (46.9)	81 (26.4)	
2021	48 (63.2)	28 (36.8)	76 (24.8)	
Type of Retainers				
Hawley Retainer	51 (56.0)	40 (44.0)	91 (29.6)	0.069
Vacuum-Formed Retainer	100 (66.7)	50 (33.3)	150 (48.9)	
Both	34 (51.6)	32 (48.5)	66 (21.5)	

ªFisher’s exact test.

*Significant difference at *P* < 0.05.

Males were shown to have greater compliance with their retainers than females; however, this association was not significant (Table 1). It was reported that the youngest age group was the most compliant, with 63.3% of them wearing their retainers as instructed by their orthodontist. Employed respondents were the most compliant while self-employed respondents were the least compliant. Those with secondary school as their highest level of education displayed greater compliance than other educational levels; however, this difference was not significant. Individuals who removed their braces in 2021 were the most compliant, while those who removed their braces in 2020 were shown to be the least compliant. Similarly, no significant association was found. It was shown that Vacuum-Formed retainers wearers (66.7%) were more compliant compared to other types of wearers.

Both retainer types showed equal mean satisfaction level values of 3.7, and the difference between them was not significant (*P* = 0.565), with one being the least happy and five being the happiest with their retainers (Table 2).

**Table 2:** Comparison of the level of satisfaction between hawley retainer and vacuum-formed retainer (Independent T-Test).

	Type of Retainers				
	Hawley Retainer		Vacuum-Formed Retainer		*P* value
	Mean	SD	Mean	SD	
Level of Satisfaction	3.7	1.0	3.7	1.1	0.565

Score range from 1 – Very unhappy to 5 – Very happy.

Majority of the respondents like wearing their retainers (Table 3). It was reported that most respondents wear their retainers to keep their teeth straight. The most common reason Hawley retainer wearers do not wear their retainers is due to difficulty in talking (32.7%), as opposed to only 11.7% among the Vacuum-Formed retainer wearers. Over a quarter of them do not like the way it feels for both retainers. A relatively high proportion of both wearers do not wear their retainers due to forgetting to wear them, at 17.3% and 19.0% for Hawley retainers and Vacuum-Formed retainers, respectively. Most of the Vacuum-Formed retainers who reported “others” revealed that they do not wear their retainers because it cracks easily.

**Table 3:** Reasons participants like and dislike using retainers.

		*n*	%
Participants like wearing Hawley Retainer	Yes	96	61.1
	No	61	38.9
Participants like wearing Vacuum-Formed Retainer	Yes	120	55.6
	No	96	44.4
Reason to use Hawley Retainer	Want to keep my teeth straight	68	28.2
	Easy to clean my teeth because it’s removable	50	20.7
	Must follow orthodontist instruction	43	17.8
	Acceptable appearance	38	15.8
	Comfortable	36	14.9
	Fashionable	3	1.2
	Others	3	1.2
Reason to use Vacuum-Formed Retainer	Want to keep my teeth straight	89	28.4
	Must follow orthodontist instruction	62	19.8
	Easy to clean my teeth because it’s removable	60	19.2
	Comfortable	48	15.3
	Acceptable appearance	46	14.7
	Others	6	1.9
	Fashionable	2	0.6
Reason for not using Hawley Retainer	It makes me hard to talk	34	32.7
	I don’t like the way it feels	28	26.9
	I forget to wear it	18	17.3
	My retainer doesn’t fit anymore	11	10.6
	I don’t like the way it looks	6	5.8
	Others	5	4.8
	I lost my retainer	2	1.9
Reason for not using Vacuum-Formed Retainer	I don’t like the way it feels	41	25.2
	I forget to wear it	31	19.0
	My retainer doesn’t fit anymore	30	18.4
	Others	30	18.4
	It makes me hard to talk	19	11.7
	I don’t like the way it looks	6	3.7
	I lost my retainer	6	3.7

*n* frequency, *%* percentage.

It was reported that most people wear their Hawley retainer full time compared to other retainer wearers (Table 4). More than half of the Hawley retainer wearers use their retainer full time (53.8%). As for Vacuum-Formed retainer wearers, it was shown that they have a wider range of actual wear time as they occupy a higher percentage in the remaining wear time except for full time. Among those Vacuum Formed retainer wearers who reported “others”, 11 said they do not wear their retainers as often or are not wearing anymore.

**Table 4:** Actual wear time according to type of retainers.

	Types of retainers						
	Hawley retainer		Vacuum-Formed Retainer		Both		*P* value
Actual Wear Time	*n*	%	*n*	%	*n*	%	
Every day/night	45	26.3	92	53.8	34	19.9	<0.001*
Alternate Days	7	24.1	22	75.9	-	-	0.005*
1–2 times per week	2	20.0	7	70.0	1	10.0	0.045*
1–2 times per month	4	26.7	3	20.0	8	53.3	0.247
Full Time	28	53.8	15	28.8	9	17.3	0.004*
Others	3	13.0	15	65.2	5	21.7	0.005*
Inconsistent wear	3	13.0	15	65.2	5	21.7	0.005*

*n* frequency, *%* percentage.

^*^Significant difference at *P* < 0.05.

Table 5 compares the actual wear time with the demographic factors. Those who reported wearing part time has been included as wearing every day at night due to their similarity. Most males and females wear their retainers every day at night, although the figure for females was slightly higher. This trend was the opposite for full-time wear, as more males than females wear their retainers full time. The proportion of people wearing their retainers every day at night decreases as the age group increases from under 20 years to 40 years. In contrast, the figures for full-time wear was higher among those under 20 years and between 30 and 40 years than those between 20 and 30 years. A significantly higher proportion of those employed and studying wear their retainers every day at night and full time. It was shown that those with secondary school as their highest level of education wear their retainers longer than others. The percentage of respondents wearing their retainers every day at night decreases as the duration of removal increases. For those who removed their braces in 2021, 61.8% of them wear their retainers every day at night, as opposed to 45.7% of them who removed their braces in 2018. However, most people who wear their retainers full time removed their retainers in 2019, followed by 2021, 2018, and 2020.

**Table 5:** Actual wear time according to demographic factors.

Demographic Factors		*n*	%	*P* value
Male	Every day/night	31	52.5	<0.001*
	Alternate day/night	5	8.5	
	Once/twice every week	1	1.7	
	Once/twice every month	4	6.8	
	Full time	12	20.3	
	Others	4	6.8	
	Inconsistent	2	3.4	
Female	Every day/night	140	56.5	<0.001*
	Alternate day/night	24	9.7	
	Once/twice every week	9	3.6	
	Once/twice every month	11	4.4	
	Full time	40	16.1	
	Others	19	7.7	
	Inconsistent	5	2.0	
<20	Everyday/night	29	59.2	<0.001*
	Alternate day/night	3	6.1	
	Once/twice every week	1	2.0	
	Once/twice every month	1	2.0	
	Full time	9	18.4	
	Others	5	10.2	
	Inconsistent	1	2.0	
20–30	Every day/night	118	55.7	<0.001*
	Alternate day/night	20	9.4	
	Once/twice every week	6	2.8	
	Once/twice every month	13	6.1	
	Full time	35	16.5	
	Others	15	7.1	
	Inconsistent	5	2.4	
30–40	Every day/night	20	48.8	<0.001*
	Alternate day/night	5	12.2	
	Once/twice every week	3	7.3	
	Once/twice every month	1	2.4	
	Full time	8	19.5	
	Others	3	7.3	
	Inconsistent	1	2.4	
>40	Every day/night	4	80.0	0.18
	Alternate day/night	1	20.0	
Employed	Every day/night	83	52.5	<0.001*
	Alternate day/night	17	10.8	
	Once/twice every week	7	4.4	
	Once/twice every month	8	5.1	
	Full time	26	16.5	
	Others	13	8.2	
	Inconsistent	4	2.5	
Unemployed	Every day/night	27	64.3	<0.001*
	Alternate day/night	3	7.1	
	Once/twice every week	1	2.4	
	Once/twice every month	2	4.8	
	Full time	7	16.7	
	Others	7	4.8	
Self-Employed	Every day/night	3	42.9	0.683
	Once/twice every week	1	14.3	
	Full time	1	14.3	
	Others	1	14.3	
	Inconsistent	1	14.3	
Studying	Every day/night	58	58.0	<0.001*
	Alternate day/night	9	9.0	
	Once/twice every week	1	1.0	
	Once/twice every month	5	5.0	
	Full time	18	18.0	
	Others	7	7.0	
	Inconsistent	2	2.0	
Secondary School	Every day/night	19	59.4	<0.001*
	Alternate day/night	3	9.4	
	Once/twice every week	1	3.1	
	Once/twice every month	1	3.1	
	Full time	6	18.8	
	Others	2	6.3	
Pre-University	Every day/night	14	50.0	0.002*
	Alternate day/night	2	7.1	
	Once/twice every month	3	10.7	
	Full time	4	14.3	
	Others	5	17.9	
Tertiary Education	Every day/night	138	55.9	<0.001*
	Alternate day/night	24	9.7	
	Once/twice every week	9	3.6	
	Once/twice every month	11	4.5	
	Full time	42	17.0	
	Others	16	6.5	
	Inconsistent	7	2.8	
2018	Every day/night	32	45.7	<0.001*
	Alternate day/night	9	12.9	
	Once/twice every week	2	2.9	
	Once/twice every month	4	5.7	
	Full time	13	18.6	
	Others	6	8.6	
	Inconsistent	4	5.7	
2019	Every day/night	42	52.5	<0.001*
	Alternate day/night	10	12.5	
	Once/twice every month	5	6.3	
	Full time	16	20.0	
	Others	6	7.5	
	Inconsistent	1	1.3	
2020	Every day/night	50	61.7	<0.001*
	Alternate day/night	9	11.1	
	Once/twice every week	7	8.6	
	Once/twice every month	3	3.7	
	Full time	8	9.9	
	Others	3	3.7	
	Inconsistent	1	1.2	
2021	Every day/night	47	61.8	<0.001*
	Alternate day/night	1	1.3	
	Once/twice every week	1	1.3	
	Once/twice every month	3	3.9	
	Full time	15	19.7	
	Others	8	10.5	
	Inconsistent	1	1.3	

*Significant difference at *P* < 0.05.

## Discussion

This study assessed the levels of retainer wear compliance among patients who have removed their braces in Brunei government orthodontic clinics and the reasons for low patient compliance. Due to the wide variety of factors, it is difficult to assess the levels of patient compliance [11]. However, compliance with removable orthodontic appliances remains essential in orthodontic treatment as it relies entirely on the patient’s responsibility for retention [7]. Therefore, it highlights the importance of this study.

Regarding the retainer types prescribed among the respondents, Vacuum-Formed Retainer (VFR) was the most popular type given to these participants. This was true in Ireland and Malaysia, where VFR was the most popular option for the lower arch [15, 16]. As for the upper arch, VFR is popular in the UK, Ireland and Malaysia [15–17]. Before, the Hawley retainer had been the most popular, but the VFR has recently gained popularity [18]. This may be due to the cheaper fabrication cost and can be done in-house in many practices [19].

In the present study, it has been shown that there was no significant difference between the levels of satisfaction of Hawley retainer and VFR. This agrees with Chagas et al. [20] where there was no difference between the overall satisfaction in the two retainer types. However, the participants recruited in this study only wore one type of retainer. This may contribute to having a similar level of satisfaction between the two retainer types due to a lack of experience in wearing the other retainer.

More respondents liked wearing their Hawley retainers. This could be reflected in their actual wear times, where a higher percentage of Hawley retainer wearers use their retainer full time while VFR wearers had a more diverse wear time. Although we did not precisely measure the preference in the different types of retainers, this result may show that Hawley retainers were more acceptable by the respondents. However, this disagrees with a study by Hichens et al. [19], where they concluded that most of their subjects preferred VFR compared with Hawley retainers.

The majority of those wearing Hawley retainer and VFR reported that they want to keep their teeth straight as a reason for wearing their retainers. This may indicate that most respondents were taught about the importance of retainers in retention. This is crucial as it is difficult to predict which patients will have a relapse after debonding; orthodontists have the responsibility to explain the unpredictability of relapse, the factors that are known to play a role, and offer advice on how to reduce the likelihood of recurrence through the proper use of retainers [21]. With that, the clinician can ensure that patients achieve optimal results from their treatment [22].

There was a considerable difference between those who reported Hawley retainers made them difficult to talk compared to VFR. This is consistent with a survey carried out by Sawhney [13], where the Hawley retainer affected speech the most in the maxilla compared to VFR. Moreover, Wan et al. [23] revealed that while sound distortion was discovered in both the Hawley and VFR groups, the Hawley group’s speech articulation modifications were more noticeable.

Many respondents also reported that they dislike how it feels and being forgetful. Similar findings were reported by, Wong and Freer [24] where discomfort and forgetfulness were the major reasons for not wearing a retainer. An important feature to note is the relatively high number of VFR wearers reported that their retainer is more prone to breakages as their other reasons for not wearing their retainers. This agrees with Manzon et al. [25], who found that VFR had a greater minor and major breakage rate than Hawley retainers. This finding may contribute to the reason why Hawley retainers are more acceptable.

Regarding the proportion of respondents who wear their retainers as instructed by their orthodontists (compliant), there was no significant difference between Hawley retainers and VFR. This is similar to several studies where no significant difference between the retainer types and compliance was noted [9, 10, 13]. This may be attributed to the small sample size and the short range of year since debond of fixed appliance, as it was reported to be the opposite in another study which concluded that patient compliance with VFR was initially higher but declined at a faster rate than with Hawley retainers, resulting in an overall higher compliance with Hawley retainers [18].

It was found that the compliance level was higher in males than females, more males also wear their retainers full time than females. However, this difference was not significant and was consistent with several studies [7, 13] This may be due to the small number of male respondents; the recruited males may be the ones who were compliant, therefore, showing a higher percentage of compliance.

Regarding the age groups, the highest proportion of respondents who wears their retainers every day at night was from the youngest age group. In addition, this age group showed a relatively high proportion of people wearing their retainers full time compared to other age groups, just slightly less than the figure of those aged 30–40 years. Moreover, it was reported that the youngest age group was the most compliant. Although most studies reported no significant association between age and compliance [9, 13], one possible reasons may be because younger children are more obedient to their orthodontist’s instructions than older people.

Regarding the employment status, respondents who were employed during the study period showed to be the most compliant. This particular demographic factor has not been studied in other articles. A possible explanation for this finding is that those employed are more aware of the consequences of relapse; the additional time and money needed for further treatment, which may affect their work.

When comparing the highest level of education and the time since the removal of braces, both showed no significant difference with the compliance. However, this is not in agreement with other studies as it was reported that the compliance decreased as the time since debond increased [10, 18]. This may not be shown in our study due to the small sample size, and only four years of patients were recruited.

Therefore, clinicians’ attitudes may play an important role; they should educate patients on the importance of wearing their retainers as most people reported that they want to keep their teeth straight as their reason to wear their retainers. Moreover, the shape of the retainers may be modified to allow better speech. Lastly, clinicians may prescribe the type of retainers according to patients’ preferences, such as durability or aesthetics.

## Limitations and recommendations

Several limitations have been identified in this study. Firstly, this was a self-reported survey which may contribute to recall bias. Also, the small sample size as the minimum target sample size was not reached, which may lead to type II errors in the results of statistical tests. As for the recommendation for future research, hardcopy questionnaires can be used, and online forms can be distributed in more media platforms, also participants can be contacted through telephone calls to increase the response rate and better understand the questionnaire.

## Conclusion

The study concluded that there was no significant difference between the satisfaction levels for Hawley retainer and Vacuum-Formed retainer. The majority of respondents wear their retainers to keep their teeth straight. The Hawley retainer group experienced speech difficulties more than the Vacuum-formed retainer group. Also, comfort and forgetfulness were the primary reasons for not wearing retainers, besides difficulty in speech. Age and employment status were the two variables that determined compliance.

## Supplementary information

Appendix 1
