# Supplementary material for: A survey of patient compliance with removable orthodontic retainer wear in Brunei Darussalam
Source: BDJ Open. 2023 Mar 3;9:10. doi: 10.1038/s41405-023-00138-8 (PMC9984364; doi:10.1038/s41405-023-00138-8)
Supplement: Supplementary file 1 — Appendix 1 [file 41405_2023_138_MOESM1_ESM.pdf]

Questionnaire

1. Gender

☐ Male

☐ Female

2. Age

____________ years

3. Are you currently ____?

☐ Employed

☐ Unemployed

☐ Self-employed

☐ Studying

4. What is the highest level of education you have completed? If you are currently studying, what level of education are you on?

☐ Secondary school

☐ Pre-University

☐ Tertiary education (E.g. University abroad, UBD, UTB, IBTE, Polytechnic Brunei, UNISSA, LCB, IGS, KUPUSB)

5. Which year were your braces removed?

☐ 2018

☐ 2019

☐ 2020

☐ 2021

6. What type of **upper** removable retainer are you wearing?

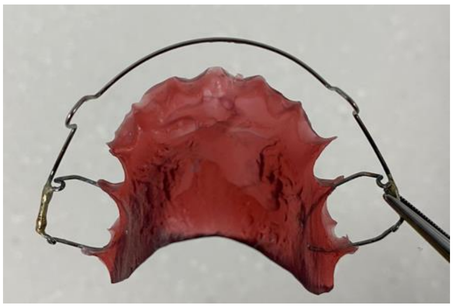

☐ Hawley Retainer

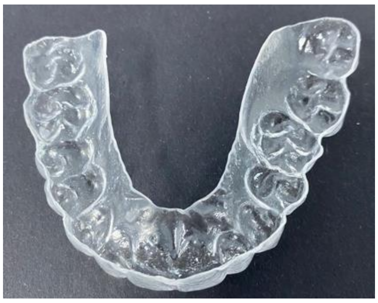

☐ Vacuum-Formed Retainer

7. What type of **lower** removable retainer are you wearing?

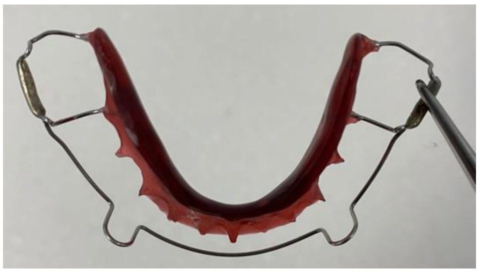

☐ Hawley Retainer

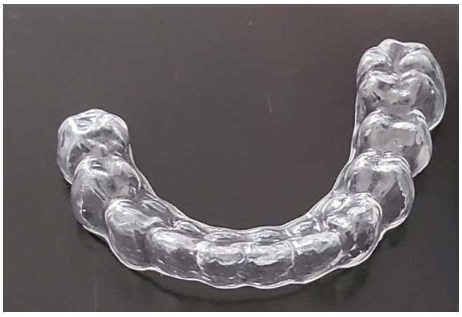

☐ Vacuum-Formed Retainer

8. How often were you instructed to wear your retainer(s)?

☐ Everyday/night

☐ Alternate day/night

☐ Once/twice every week

☐ Once/twice every month

☐ Full time (Both day and night, except during BRUSHING OR EATING)

☐ Part time (Night only or day only, AT LEAST 10 HOURS A DAY)

☐ Others:_____

9. How often do you wear your retainer(s)?

☐ Everyday/night

☐ Alternate day/night

☐ Once/twice every week

☐ Once/twice every month
☐ Full time (Both day and night, except during BRUSHING OR EATING)
☐ Part time (Night only or day only, AT LEAST 10 HOURS A DAY)
☐ Others:_____

10. Please tick one of the following. How satisfied are you with your Hawley retainer? *(This question will only be displayed if participant chose Hawley retainer for Q6 and/or Q7)*

☐ Very unhappy
☐ Somewhat unhappy
☐ Neither unhappy nor happy
☐ Somewhat happy
☐ Very happy
☐ Do not know

11. Please tick one of the following. How satisfied are you with your Vacuum-Formed retainer? *(This question will only be displayed if participant chose Vacuum-Formed retainer for Q6 and/or Q7)*

☐ Very unhappy
☐ Somewhat unhappy
☐ Neither unhappy nor happy
☐ Somewhat happy
☐ Very happy
☐ Do not know

12. Do you like wearing your Hawley retainer? *(This question will only be displayed if participant chose Hawley retainer for Q6 and/or Q7)*

☐ YES
☐ NO

13. Do you like wearing your Vacuum-Formed retainer? *(This question will only be displayed if participant chose Vacuum-Formed retainer for Q6 and/or Q7)*

☐ YES
☐ NO

14. Why do you like wearing your Hawley retainer(s)? (check all that apply) *(Will only be displayed if participant chose Hawley retainer for Q6 and/or Q7, and if participant chose 'YES' for Q12)*

- ☐ Comfortable
- ☐ Acceptable appearance
- ☐ Easy to clean my teeth because its removable
- ☐ Want to keep my teeth straight
- ☐ Must follow orthodontist instruction
- ☐ Fashionable
- ☐ Others ___________________

15. Why do you like wearing your Vacuum-Formed retainer(s)? (check all that apply) *(Will only be displayed if participant chose Vacuum-Formed retainer for Q6 and/or Q7, and it participant chose 'YES' for Q13)*

- ☐ Comfortable
- ☐ Acceptable appearance
- ☐ Easy to clean my teeth because its removable
- ☐ Want to keep my teeth straight
- ☐ Must follow orthodontist instruction
- ☐ Fashionable
- ☐ Others ___________________

16. If you are not wearing your Hawley retainer(s) as often as you were instructed, which of the following contributes to this difference? (Check all that apply) *(Will only be displayed if participant chose Hawley retainer for Q6 and/or Q7, and if participant chose 'NO' for Q12)*

- ☐ I don't like the way it feels
- ☐ I don't like the way it looks
- ☐ I forget to wear it
- ☐ I lost my retainer
- ☐ My retainer doesn't fit anymore
- ☐ It makes me hard to talk
- ☐ Others: __________

17. If you are not wearing your Vacuum-Formed retainer(s) as often as you were instructed, which of the following contributes to this difference? (Check all that apply) *(Will only be*

displayed if participant chose Vacuum-Formed retainer for Q6 and/or Q7, and if participant chose ‘NO’ for Q13)

- ☐ I don’t like the way it feels
- ☐ I don’t like the way it looks
- ☐ I forget to wear it
- ☐ I lost my retainer
- ☐ My retainer doesn’t fit anymore
- ☐ It makes me hard to talk
- ☐ Others: __________
